# Molecular phylogeny of *Atractus* (Serpentes, Dipsadidae), with emphasis on Ecuadorian species and the description of three new taxa

**DOI:** 10.3897/zookeys.661.11224

**Published:** 2017-03-15

**Authors:** Alejandro Arteaga, Konrad Mebert, Jorge H. Valencia, Diego F. Cisneros-Heredia, Nicolás Peñafiel, Carolina Reyes-Puig, José L. Vieira-Fernandes, Juan M. Guayasamin

**Affiliations:** 1 Tropical Herping, Av. 6 de Diciembre N31-50 y Whymper. Torres Santa Fe, Quito, Ecuador; 2 Universidade Estadual de Santa Cruz, Departamento de Ciências Biológicas, Rodovia Jorge Amado, Km 16, 45662-900 Salobrinho, Ilhéus, Bahia, Brazil; 3 Fundación Herpetológica Gustavo Orcés, Av. Amazonas 3008 y Calle Rumipamba, Casilla Postal 17-033-448, Quito, Ecuador; 4 Universidad San Francisco de Quito (USFQ), Colegio de Ciencias Biológicas y Ambientales, Laboratorio de Biología Evolutiva, Laboratorio de Zoología Terrestre, campus Cumbayá, Casilla Postal 17-1200-841, Quito, Ecuador; 5 Centro de Investigación de la Biodiversidad y Cambio Climático (BioCamb), Facultad de Ciencias de Medio Ambiente, Ingeniería en Biodiversidad y Recursos Genéticos, Universidad Tecnológica Indoamérica, Av. Machala y Sabanilla, Quito, Ecuador; 6 Museo Ecuatoriano de Ciencias Naturales, Instituto Nacional de Biodiversidad, División de Herpetología, calle Rumipamba 341 y Av. de los Shyris, Casilla 17-07-8976, Quito, Ecuador

**Keywords:** Pacific lowlands, biodiversity, Ecuador, groundsnakes, *Atractus*, phylogeny, new species

## Abstract

We present a molecular phylogeny of snake genus *Atractus*, with an improved taxon sampling that includes 30 of the 140 species currently recognized. The phylogenetic tree supports the existence of at least three new species in the Pacific lowlands and adjacent Andean slopes of the Ecuadorian Andes, which we describe here. A unique combination of molecular, meristic and color pattern characters support the validity of the new species. With the newly acquired data, we propose and define the *Atractus iridescens* species group, as well as redefine the *Atractus roulei* species group. The species *Atractus iridescens* is reported for the first time in Ecuador, whereas *Atractus bocourti* and *Atractus medusa* are removed from the herpetofauna of this country. We provide the first photographic vouchers of live specimens for *Atractus multicinctus*, *Atractus paucidens* and *Atractus touzeti*, along with photographs of 19 other Ecuadorian *Atractus* species. The current status of *Atractus occidentalis* and *Atractus paucidens* is maintained based on the discovery of new material referable to these species. With these changes, the species number reported in Ecuador increases to 27, a number that is likely to increase as material not examined in this work becomes available and included in systematic studies.

## Introduction

With 140 species, *Atractus* is the most speciose snake genus in the world, with 33 new species described only during the last ten years (Uetz et al. 2016). Most of these new species have been described using a combination of meristic and morphometric characters (Passos et al. 2009a, 2016, Passos and Lynch 2010, Schargel et al. 2013, Salazar-Valenzuela et al. 2014). However, with the exception of the preliminary phylogeny presented in De Oliveira and Hernández-Ruz (2016), no studies have involved a phylogenetic approach to test species arrangements and boundaries.

One recent work by Passos et al. (2009a) evaluated the taxonomic status of *Atractus* species from the Pacific lowland of Colombia and Ecuador, using a combination of meristic, morphometric, color pattern, and hemipenial characters. These authors described three new species and provided a comprehensive review of all *Atractus* known to occur in the region. However, when referring to this work to compare previously unexamined material from Ecuador, it became clear to us that several Ecuadorian specimens of Pacific lowland *Atractus* could not be assigned to any taxa currently recognized to occur in the country. Some specimens identified as *Atractus medusa* (Passos et al. 2009a) matched the coloration of the first specimen reported in Ecuador by Cisneros-Heredia and Romero (2015), but they did not match the coloration of the holotype (Passos et al. 2009a). Other specimens were closer in coloration and lepidosis to *Atractus iridescens* (Peracca, 1860) from Colombia, and others resembled both *Atractus microrhynchus* (Cope, 1868) and *Atractus occidentalis* (Savage, 1955). To further complicate matters, the taxonomic validity of *Atractus occidentalis* and *Atractus paucidens* (Despax, 1910) was not recognized in Arteaga et al. (2013), owing to their close morphological resemblance to *Atractus dunni* (Savage, 1955) and *Atractus modestus* (Boulenger, 1894), respectively.

To resolve these pending issues and to shed light on potentially unclear species boundaries, we report on new material of *Atractus* from Ecuador, review current knowledge on the species occurring in the Pacific lowlands and adjacent Andean slopes, present a new molecular phylogeny, including most Ecuadorian species, and describe three new species of *Atractus*.

## Materials and methods

### Ethics statement

This study was carried out in strict accordance with the guidelines for use of live amphibians and reptiles in field research compiled by the American Society of Ichthyologists and Herpetologists (ASIH), The Herpetologists’ League (HL) and the Society for the Study of Amphibians and Reptiles (SSAR). All procedures with animals (see below) were approved by the Centro de Investigación de la Biodiversidad y Cambio Climático (BioCamb) of the Universidad Tecnológica Indoamérica. They also were reviewed by the Ministerio de Ambiente del Ecuador (MAE) and specifically approved as part of obtaining the following field permits for research and collection: MAE-DNB-CM-2015-0017, granted to Universidad Tecnológica Indoamérica; and permit N°012-IC-FAN-DPEO-MAE, granted to the Museo Ecuatoriano de Ciencias Naturales. Specimens were euthanized with 20% benzocaine, fixed in 10% formalin or 70% ethanol, and stored in 70% ethanol. Museum vouchers were deposited at the Museo de Zoología of the Universidad Tecnológica Indoamérica (MZUTI).

### Sampling

Tissue samples from 39 individuals representing 22 species (including three new species described here) were obtained throughout Ecuador. The majority of individuals were located by space-constrained visual examination of ground-level substrates (Campbell and Christman 1982). The remaining individuals were detected by turning over logs, rocks and other surface objects. All specimens included in the genetic analyses were morphologically identified according to Savage (1955, 1960), Cisneros-Heredia (2005), Passos et al. (2009a), Arteaga et al. (2013), Schargel et al. (2013) and Salazar-Valenzuela et al. (2014). We generated sequence data for samples marked with an asterisk under Appendix I, which includes museum vouchers at the Museo de Zoología de la Universidad Tecnológica Indoamérica (MZUTI), the División de Herpetología del Museo Ecuatoriano de Ciencias Naturales (DHMECN) and the Fundación Herpetológica Gustavo Orcés (FHGO).

### Laboratory techniques

Genomic DNA was extracted from 96% ethanol-preserved tissue samples (liver, muscle tissue or scales) using a modified salt precipitation method based on the Puregene DNA purification kit (Gentra Systems). We amplified the 16S gene using the primers 16Sar-L and 16Sbr-H-R from Palumbi et al. (1991). Additionally, the Cytb gene was obtained with the primers L14910 and H16064 developed by Burbrink et al. (2000), whereas the gene coding for the subunit 4 of the NADH dehydrogenase was amplified with the primers ND4 and Leu developed by Arévalo et al. (1994). PCR reactions contained 2 mM (Cytb and ND4) or 3 mM (16S) MgCl_2_, 200 µM dNTP mix, 0.2 µM (16S and Cytb) or 0.8 µM (ND4) of each primer and 1.25 U (16S and Cytb) or 0.625 U (ND4) Taq DNA Polymerase Recombinant (Thermo Fisher Scientific) in a 25 µL total volume. The nucleotide sequences of the primers and the PCR conditions applied to each primer pair are detailed in Appendix II. PCR products were cleaned with Exonuclase I and Alkaline Phosphatase (Illustra ExoProStar by GE Healthcare) before they were sent to Macrogen Inc (Korea) for sequencing. All PCR products were sequenced in both forward and reverse directions with the same primers that were used for amplification. The edited sequences were deposited in GenBank (Appendix I).

### DNA sequence analyses

A total of 126 mtDNA sequences were used to build a mitochondrial phylogenetic tree of the genus *Atractus*. 69 were generated during this work and 57 (all available sequences for the sampled gene fragments) were downloaded from GenBank. A mitochondrial marker dataset, though less powerful to study higher-level phylogenetic relationships, was chosen because it is the most effective to successfully resolve species-level phylogenies (Patwardhan 2014). Recently published works looking to resolve intrageneric relationships within Neotropical dipsadines have done so using phylogenies that are largely based on mitochondrial data (Krysko et al. 2015, Pyron et al. 2016). Specifically, we use the gene Cytochrome-b because it is reported as the most powerful in recovering phylogenetic relationships among closely related taxa (Patwardhan 2014), which is the case for the species of *Atractus* studied here. The mitochondrial genes 16S and ND4 were used to be able to compare with *Atractus* sequences available in GenBank. Novel sequences were edited and assembled using the program Geneious ProTM 5.4.7 (Drummond et al. 2010) and aligned with those downloaded from Genbank (Appendix I) using MAFFT v.7 (Katoh and Standley 2013) under the default parameters in Geneious ProTM 5.4.7. Genes were combined into a single matrix with seven partitions, one per non-coding gene and three per protein coding gene corresponding to each codon position. The best partition strategies along with the best-fit models of evolution were obtained in PartitionFinder 1.1.1 (Lanfear et al. 2012) and jModeltest (Darriba et al. 2012) under the Bayesian information criterion. Phylogenetic relationships were assessed under a Bayesian approach in MrBayes 3.2.0 (Ronquist and Huelsenbeck 2013). Four independent analyses were performed to reduce the chance of converging on a local optimum. Each analysis consisted of 6.7 million generations and four Markov chains with default heating settings. GenBank accession numbers are listed in Appendix I. Trees were sampled every 1,000 generations, resulting in 5,000 saved trees per analysis after 25% of those were arbitrarily discarded as ‘‘burn-in.” Stationarity was confirmed by plotting the–ln L per generation in the program Tracer 1.2 (Rambaut and Drummond 2003). Genetic distances between *Atractus esepe* and its closest morphological relatives were calculated using the uncorrected distance matrix in PAUP 4.0 (Swofford 2002).

### Morphological data

Our terminology for *Atractus* cephalic shields follows Savage (1960), diagnoses and descriptions generally follow Passos et al. (2009a), and ventral and subcaudal counts follow Dowling (1951). We examined comparative alcohol-preserved specimens from the herpetology collections at the MZUTI, DHMECN, Fundación Herpetológica Gustavo Orcés (FHGO), Museum d’Histoire Naturelle de la Ville de Genève (MHNG), Museo de Historia Natural de la Escuela Politécnica Nacional (EPN), Museo de Zoología de la Pontificia Universidad Católica del Ecuador (QCAZ), National Museum of Natural History (USNM), Muséum National d’Histoire Naturelle (MNHN) and Museo de Zoología de la Universidad San Francisco de Quito (ZSFQ). (Table 1). Morphological measurements were taken with measuring tapes to the nearest 1 mm. When providing the standard deviation, we use the ± symbol. Sex was determined by noting the presence or absence of hemipenes through a subcaudal incision at the base of the tail.

**Table 1.** Locality data for specimens examined in this study. Coordinates represent georeferencing attempts from gazetteers under standard guidelines, though some variation from the exact collecting locality will be present. Similarly, elevations are taken from Google Earth, and may not exactly match the elevations as originally reported.

Species	Voucher	Locality	Latitude	Longitude	Elev.
*Atractus carrioni*	DHMECN 4697	Loja, Utuana	-4.36642	-79.72483	2517
*Atractus carrioni*	DHMECN 76	Esmeraldas, Copa Quininde (in error)	0.06181	-78.72641	1688
*Atractus carrioni*	DHMECN 7668	Loja, Utuana	-4.36642	-79.72483	2517
*Atractus carrioni*	MZUTI 4194	Loja, Utuana	-4.36642	-79.72483	2517
*Atractus carrioni*	MZUTI 4195	Loja, Utuana	-4.36642	-79.72483	2517
*Atractus duboisi*	MHNG 2457.093	Napo, Chiriboga (in error)	-	-	-
*Atractus duboisi*	MNHN 0.6147	Ecuador	-	-	-
*Atractus duboisi*	MZUTI 3640	Napo, Yanayacu	-0.60071	-77.88927	1924
*Atractus duboisi*	MZUTI 62	Napo, Yanayacu	-0.59939	-77.89050	2064
*Atractus dunni*	DHMECN 12769	Carchi, Gualpi	0.86439	-78.22435	2104
*Atractus dunni*	DHMECN 2215	Pichincha, Río Cambugán	0.17697	-78.50779	1828
*Atractus dunni*	DHMECN 3527	Imbabura, Junín	0.27009	-78.64975	1688
*Atractus dunni*	DHMECN 3900	Pichincha, Tambo Quinde	0.00967	-78.66906	1870
*Atractus dunni*	DHMECN 4159	Pichincha, Pahuma	0.02757	-78.63208	1914
*Atractus dunni*	EPN 3127	Santo Domingo, Chiriboga	-0.22841	-78.76725	1813
*Atractus dunni*	EPN 3128	Santo Domingo, Chiriboga	-0.22841	-78.76725	1813
*Atractus dunni*	FHGO 375	Santo Domingo, La Favorita	-0.22833	-78.76503	1810
*Atractus dunni*	FHGO 376	Santo Domingo, La Favorita	-0.22833	-78.76503	1810
*Atractus dunni*	FHGO 379	Santo Domingo, La Favorita	-0.22833	-78.76503	1810
*Atractus dunni*	FHGO 91	Santo Domingo, La Favorita	-0.22833	-78.76503	1810
*Atractus dunni*	MHNG 2441.043	Cotopaxi, Cutzualo	-0.54497	-78.91891	1952
*Atractus dunni*	MHNG 2457.091	Santo Domingo, La Favorita	-0.22841	-78.76725	1813
*Atractus dunni*	MHNG 2464.03	Cotopaxi, Otonga	-0.41549	-79.00480	2095
*Atractus dunni*	MZUTI 2189	Pichincha, Tandayapa–Bellavista	-0.00843	-78.67619	1919
*Atractus dunni*	MZUTI 3031	Pichincha, Tandayapa Lodge	0.00268	-78.68131	1757
*Atractus dunni*	MZUTI 4097	Imbabura, Santa Rosa de Intag	0.37616	-78.46054	2077
*Atractus dunni*	MZUTI 4098	Imbabura, Santa Rosa de Intag	0.37616	-78.46054	2077
*Atractus dunni*	MZUTI 4099	Imbabura, Santa Rosa de Intag	0.37616	-78.46054	2077
*Atractus dunni*	MZUTI 4100	Imbabura, Below of Siempre Verde	0.37782	-78.46901	1974
*Atractus dunni*	MZUTI 4318	Imbabura, Toisán	0.53297	-78.52924	2286
*Atractus dunni*	MZUTI 4319	Imbabura, Toisán	0.53297	-78.52924	2286
*Atractus dunni*	ZSFQ 1513	Santo Domingo, Guajalito	-0.22875	-78.82248	1801
*Atractus ecuadorensis*	DHMECN 5101	Tungurahua, Río Verde	-1.40344	-78.30099	1507
*Atractus elaps*	DHMECN 10179	Morona Santiago, Tundayme	-3.57244	-78.46982	790
*Atractus gaigeae*	MHNG 2397.044	Morona Santiago, Macas	-2.31670	-78.11670	972
*Atractus gigas*	MHNG 2250.035	Santo Domingo, Chiriboga	-0.22841	-78.76725	1813
*Atractus gigas*	MHNG 2441.02	Cotopaxi, Otonga	-0.41549	-79.00480	2095
*Atractus gigas*	MZUTI 3286	Pichincha, Las Gralarias	-0.00807	-78.73238	1985
*Atractus iridescens*	DHMECN 2932	Esmeraldas, Canande	0.52993	-79.03541	594
*Atractus iridescens*	DHMECN 5663	Esmeraldas, Tundaloma	1.18236	-78.75250	74
*Atractus iridescens*	DHMECN 9633	Esmeraldas, Canande	0.52993	-79.03541	594
*Atractus iridescens*	EPN 13920	Carchi, Río Blanco	1.18993	-78.50413	223
*Atractus iridescens*	FHGO 10443	Esmeraldas, Tsejpi	0.79930	-78.84527	152
*Atractus iridescens*	MZUTI 3548	Esmeraldas, Tundaloma	1.18166	-78.74945	74
*Atractus iridescens*	MZUTI 3680	Esmeraldas, Tundaloma	1.18166	-78.74945	74
*Atractus iridescens*	MZUTI 4178	Pichincha, Puerto Quito	0.11667	-79.26661	143
*Atractus iridescens*	MZUTI 4697	Esmeraldas, Canande	0.52993	-79.03541	594
*Atractus iridescens*	ZSFQ 191.101109	Esmeraldas, Tundaloma	1.18166	-78.74945	74
*Atractus lehmanni*	DHMECN 7644	Azuay, Reserva Yunguilla	-3.22684	-79.27520	1748
*Atractus lehmanni*	DHMECN 7645	Azuay, Reserva Yunguilla	-3.22684	-79.27520	1748
*Atractus major*	ANF 1545	Orellana, Estación Científica Yasuní	-0.67781	-76.39819	246
*Atractus major*	DHMECN 8343	Sucumbíos, Bloque 27	0.32273	-76.19369	272
*Atractus major*	MNHN 0.6149	Ecuador	-	-	-
*Atractus major*	MZUTI 4973	Zamora Chinchipe, Maycu	-4.38030	-78.74584	981
*Atractus microrhynchus*	DHMECN 2536	El Oro, Buenaventura	-3.65467	-79.76794	524
*Atractus microrhynchus*	DHMECN 2586	El Oro, Buenaventura	-3.65467	-79.76794	524
*Atractus microrhynchus*	FHGO 897	El Oro, Zambo Tambo	-3.67861	-79.68001	1014
*Atractus microrhynchus*	MHNG 2307.017	El Oro, El Progreso	-3.26998	-79.73452	176
*Atractus microrhynchus*	MHNG 2397.019	El Oro, El Progreso	-3.26998	-79.73452	176
*Atractus microrhynchus*	MHNG 2397.02	El Oro, El Progreso	-3.26998	-79.73452	176
*Atractus microrhynchus*	MHNG 2397.021	El Oro, El Progreso	-3.26998	-79.73452	176
*Atractus microrhynchus*	MHNG 2459.052	El Oro, El Progreso	-3.26998	-79.73452	176
*Atractus microrhynchus*	MZUTI 4122	Manabí, Jama Coaque	-0.11556	-80.12472	299
*Atractus microrhynchus*	MZUTI 5109	Los Ríos, Río Palenque	-0.59273	-79.36369	163
*Atractus microrhynchus*	QCAZ 1219	Loja, Olmedo	-3.94994	-79.66667	1545
*Atractus microrhynchus*	USNM 285473	Los Ríos, Rio Palenque	-0.58333	-79.36667	173
*Atractus microrhynchus*	USNM 285474	Los Ríos, Rio Palenque	-0.58333	-79.36667	173
*Atractus modestus*	DHMECN 3859	El Oro, Piñas	-3.68041	-79.68253	1019
*Atractus modestus*	EPN 13916	Carchi, Chical	0.90327	-78.16201	1437
*Atractus modestus*	FHGO 2936	Pichincha, Maquipucuna	0.11757	-78.67446	1490
*Atractus modestus*	FHGO 44	Pichincha, Maquipucuna	0.11757	-78.67446	1490
*Atractus modestus*	MHNG 2397.041	Cotopaxi, Las Pampas	-0.44036	-78.96663	1590
*Atractus modestus*	MZUTI 4760	Pichincha, Gualea	0.08536	-78.74092	1557
*Atractus multicinctus*	MZUTI 5106	Esmeraldas, Canandé	0.52581	-79.2088	310
*Atractus occidentalis*	EPN 13077	Pichincha, Mindo	-0.04872	-78.77520	1277
*Atractus occidentalis*	FHGO 385	Santo Domingo, La Favorita	-0.22833	-78.76503	1810
*Atractus occidentalis*	MHNG 2252.079	Cotopaxi, Las Pampas	-0.44036	-78.96663	1590
*Atractus occidentalis*	MHNG 2307.068	Pichincha, Tandapi	-0.41522	-78.79728	1455
*Atractus occidentalis*	MHNG 2397.028	Cotopaxi, Las Pampas	-0.44036	-78.96663	1590
*Atractus occidentalis*	MHNG 2411.085	Pichincha, Tandapi	-0.41522	-78.79728	1455
*Atractus occidentalis*	MHNG 2411.086	Pichincha, Tandapi	-0.41522	-78.79728	1455
*Atractus occidentalis*	MHNG 2441.044	Pichincha, Nanegalito	0.06181	-78.72641	1688
*Atractus occidentalis*	MZUTI 1385	Pichincha, Yellow House	-0.04492	-78.75843	1504
*Atractus occidentalis*	MZUTI 2649	Pichincha, Yellow House	-0.05199	-78.76923	1325
*Atractus occidentalis*	MZUTI 2650	Pichincha, Yellow House	-0.04371	-78.75351	1520
*Atractus occidentalis*	MZUTI 3323	Pichincha,Las Gralarias	-0.00615	-78.73381	1985
*Atractus paucidens*	DHMECN 11980	Pichincha, Pedro Vicente Maldonado	0.05361	-78.92109	938
*Atractus paucidens*	DHMECN 3975	Santa Elena, Comuna Loma Alta	-1.83442	-80.70291	72
*Atractus paucidens*	EPN 8729	Santo Domingo, Finca La Esperanza	-0.27160	-79.10568	616
*Atractus paucidens*	EPN 8730	Santo Domingo, Finca La Esperanza	-0.27160	-79.10568	616
*Atractus paucidens*	EPN 8731	Santo Domingo, Finca La Esperanza	-0.27160	-79.10568	616
*Atractus paucidens*	EPN 8732	Santo Domingo, Finca La Esperanza	-0.27160	-79.10568	616
*Atractus paucidens*	MHNG 2309.065	Pichincha, Puerto Quito	0.11667	-79.26661	143
*Atractus paucidens*	MNHN 1906.245	Santo Domingo, Santo Domingo	-0.25351	-79.17297	554
*Atractus paucidens*	MZUTI 5102	Pichincha, Río Cinto	-0.09070	-78.80299	1409
*Atractus paucidens*	MZUTI 5104	El Oro, Buenaventura	-3.65467	-79.76794	524
*Atractus paucidens*	MZUTI 5105	Pichincha, Río Cinto	-0.09070	-78.80299	1409
*Atractus resplendens*	MZUTI 3996	Tungurahua, Puntzan	-1.41359	-78.40951	1962
*Atractus roulei*	MZUTI 4503	Chimborazo, Vicinity of Tixán	-2.16174	-78.81227	2892
*Atractus roulei*	MZUTI 4544	Chimborazo, Vicinity of Tixán	-2.16174	-78.81227	2892
*Atractus roulei*	QCAZ 6256	Azuay, Hierba Mala	-2.76439	-79.43816	3029
*Atractus roulei*	QCAZ 7887	El Oro, Guanazán	-3.44139	-79.49417	2596
*Atractus roulei*	QCAZ 7902	El Oro, Guanazán	-3.44139	-79.49417	2596
*Atractus roulei*	QCAZ 9643	El Oro, Guanazán	-3.44139	-79.49417	2596
*Atractus roulei*	QCAZ 9652	El Oro, Guanazán	-3.44139	-79.49417	2596
*Atractus savagei*	DHMECN 3800	Carchi, Río la Plata	0.82381	-78.04584	2256
*Atractus savagei*	MZUTI 4916	Carchi, Chilma Bajo	0.86495	-78.04978	2058
*Atractus snethlageae*	MNHN 1906.244	Morona Santiago, Gualaquiza	-3.39914	-78.57859	835
*Atractus snethlageae*	MNHN 1994.1171	Morona Santiago, Gualaquiza	-3.39914	-78.57859	835
*Atractus touzeti*	ANF 2390	Pastaza, Tzarentza	-1.35696	-78.05814	1355
*Atractus trilineatus*	MNHN 1898.313	Imbabura, Paramba (in error)	0.81671	-78.35002	698
*Atractus trilineatus*	MNHN 1898.314	Imbabura, Paramba (in error)	0.81671	-78.35002	698
*Atractus typhon*	DHMECN 9632	Esmeraldas, Canandé	0.52993	-79.03541	594
*Atractus typhon*	FHGO 10438	Esmeraldas, Gualpi	0.78173	-79.15993	63
*Atractus typhon*	FHGO 10439	Esmeraldas, Gualpi	0.78173	-79.15993	63
*Atractus typhon*	MZUTI 3284	Esmeraldas, Itapoa	0.51307	-79.13400	321

### Nomenclatural acts

The electronic edition of this article conforms to the requirements of the amended International Code of Zoological Nomenclature, and hence the new names contained herein are available under that Code from the electronic edition of this article. This published work and the nomenclatural acts it contains have been registered in ZooBank, the online registration system for the ICZN. The ZooBank LSIDs (Life Science Identifiers) can be resolved and the associated information viewed through any standard web browser by appending the LSID to the prefix “http://zoobank.org/”. The LSID for this publication is: urn:lsid:zoobank.org:pub:7CBF7FB1-EFEA-4DC1-8F64-5BF862694AA0. The electronic edition of this work was published in a journal with an ISSN, and has been archived and is available from the following digital repositories: PubMed Central, LOCKSS.

## Results

### Molecular phylogeny

**Figure 1.**
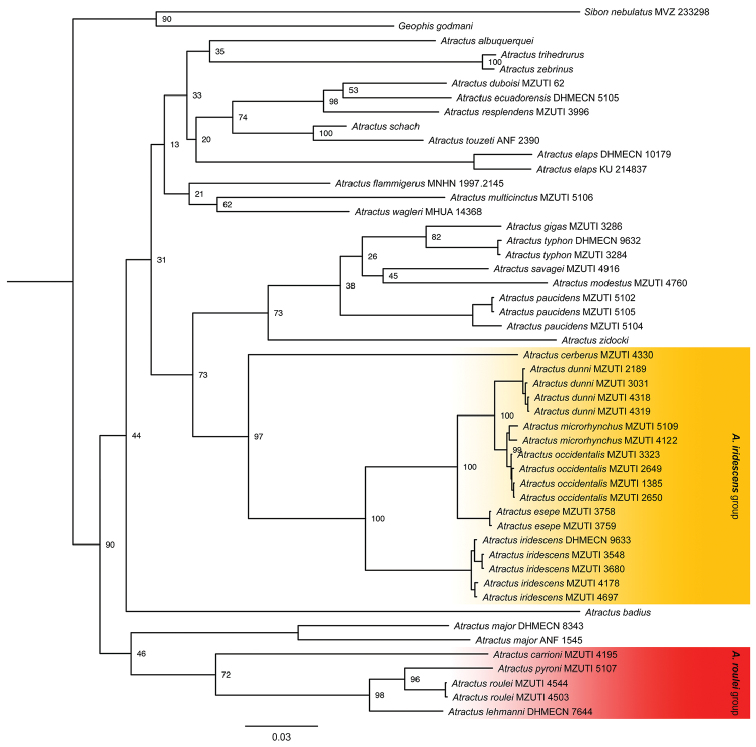
Bayesian consensus phylogeny depicting relationships within colubrid snakes of the genus *Atractus*, summarized from 5 million post-burnin generations in MrBayes 3.2.0. The topology was derived from analysis of 2,564 bp of mitochondrial DNA (gene fragments 16S, Cytb and ND4). Numbers next to branches correspond to posterior probability values. PP values on intraspecific branches are not shown for clarity. Voucher numbers for sequences are indicated for each terminal when available.

The overall topology and support (Fig. 1) is similar to that of Pyron et al. (2015). We consider strong support to be posterior probability values >95%, following Felsenstein (2004). Overall, there is low support for many backbone nodes. Strong support was found for the clade colored in yellow under Fig. 1.

The resulting hypotheses of species relationships for our mitochondrial phylogenetic tree supports Savage’s (1960) assumption suggesting independent evolution of the 15 dorsal scale row lineage within *Atractus*, since species with this number of dorsal scale rows, like *Atractus elaps*, *Atractus roulei* and *Atractus duboisi*, belong to different lineages. However, the tree does show that *Atractus carrioni* (Parker 1930), *Atractus lehmanni* (Boettger 1898), *Atractus roulei* (Despax, 1910) and *Atractus pyroni* sp. n., species with 15 scale rows, form a monophyletic group that includes two more species than was suggested by Passos et al. (2013) when naming the *Atractus roulei* species group (Fig. 1).

*Atractus gigas* (Myers and Schargel, 2006), *Atractus modestus*, *Atractus paucidens*, *Atractus savagei* (Salazar-Valenzuela et al. 2014), *Atractus typhon* (Passos et al., 2009a) and *Atractus zidoki* (Gasc and Rodrigues, 1979) form a poorly supported clade that does not include *Atractus microrhynchus* and *Atractus iridescens*, as was suggested by Passos et al. (2009a) when naming the *Atractus paucidens* species group (Fig. 1). Six species, *Atractus cerberus* sp. n., *Atractus dunni*, *Atractus esepe* sp. n., *Atractus iridescensAtractus microrhynchus*, and *Atractus occidentalis*, form a strongly supported clade sister to the *Atractus paucidens* species group. Here, we name this lineage as the *Atractus iridescens* species group (Fig. 1).

*Atractus occidentalis* forms a strongly supported distinct lineage, sister to *Atractus microrhynchus*. Together, these two species are sister to *Atractus dunni*. *Atractus typhon* is shown to be the strongly supported sister lineage of *Atractus gigas*, as is the case for a relationship between *Atractus roulei* and *Atractus pyroni* sp. n.

### New taxa and systematic arrangements derived from the analyses

We seek here to only name or redelimit *Atractus* species groups that are supported in our molecular phylogeny and share features of their coloration pattern and lepidosis. The first such groups is the clade comprising *Atractus cerberus* sp. n., *Atractus dunni*, *Atractus esepe* sp. n., *Atractus iridescens*, *Atractus microrhynchus* and *Atractus occidentalis*. The other is the one comprising *Atractus carrioni*, *Atractus lehmanni*, *Atractus pyroni* sp. n. and *Atractus roulei*.

### *Atractus iridescens* species group

**Figure 2.**
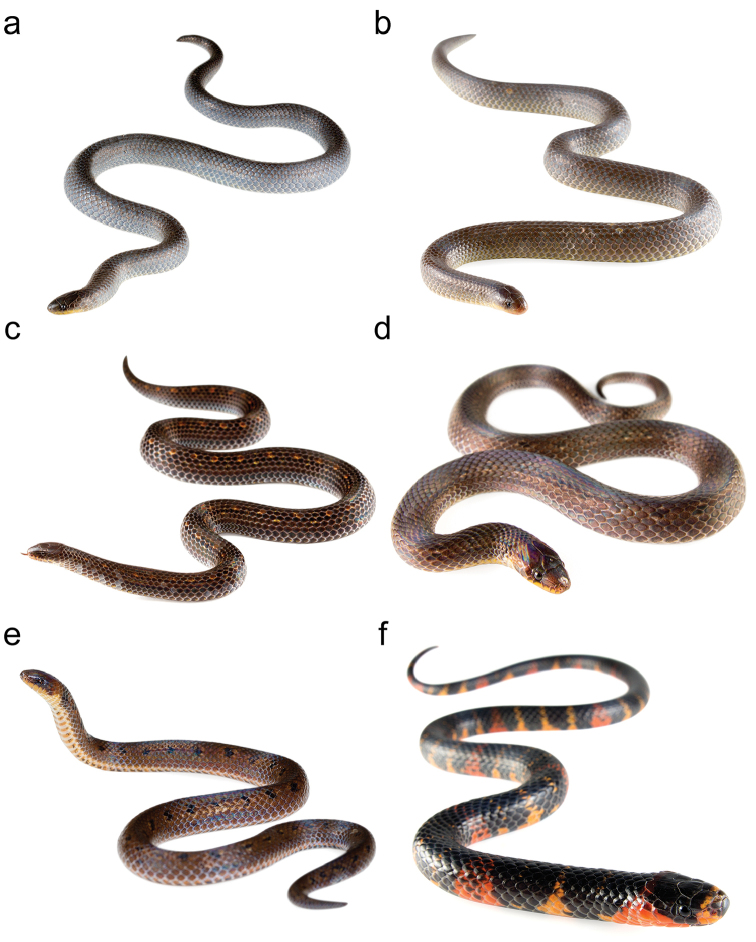
Photographs of some Ecuadorian species of *Atractus* in life: *Atractus carrioni* MZUTI 4194 (**a**), MZUTI 4195 (**b**), *Atractus duboisi* MZUTI 3640 (**c**), *Atractus dunni* MZUTI 4318 (**d**), *Atractus dunni* MZUTI 2189 (**e**), *Atractus elaps* AMARU SN (**f**), *Atractus gigas* MZUTI 3286 (**g**), *Atractus iridescens* MZUTI 3680 (**h**), *Atractus iridescens* QCAZ 8072 (**i**), *Atractus iridescens* MZUTI 4697 (**j**), *Atractus iridescens* MZUTI 3548 (**k**), *Atractus major* MZUTI 4973 (**l**), *Atractus microrhynchus* MZUTI 5109 (**m**), *Atractus modestus* (**n**), *Atractus multicinctus* MZUTI 5106 (**o**), *Atractus occidentalis* MZUTI 1385 (**p**), *Atractus occidentalis* MZUTI 3323 (**q**), *Atractus paucidens* MZUTI 5102 (**r**), *Atractus resplendens* MZUTI 3996 (**s**), *Atractus roulei* MZUTI 4503 (**t**), *Atractus savagei* MZUTI 4916 (**u**), *Atractus snethlageae* (**v**), *Atractus touzeti* ANF 2390 (**w**), and *Atractus typhon* MZUTI 5110.

**Diagnosis.** 200–360 mm SVL *Atractus* with brown dorsal ground color bearing a pattern of dots or stripes (Fig. 2), generally 17/17/17 smooth dorsals, and 125–163 ventrals (Table 2).

**Figure:**
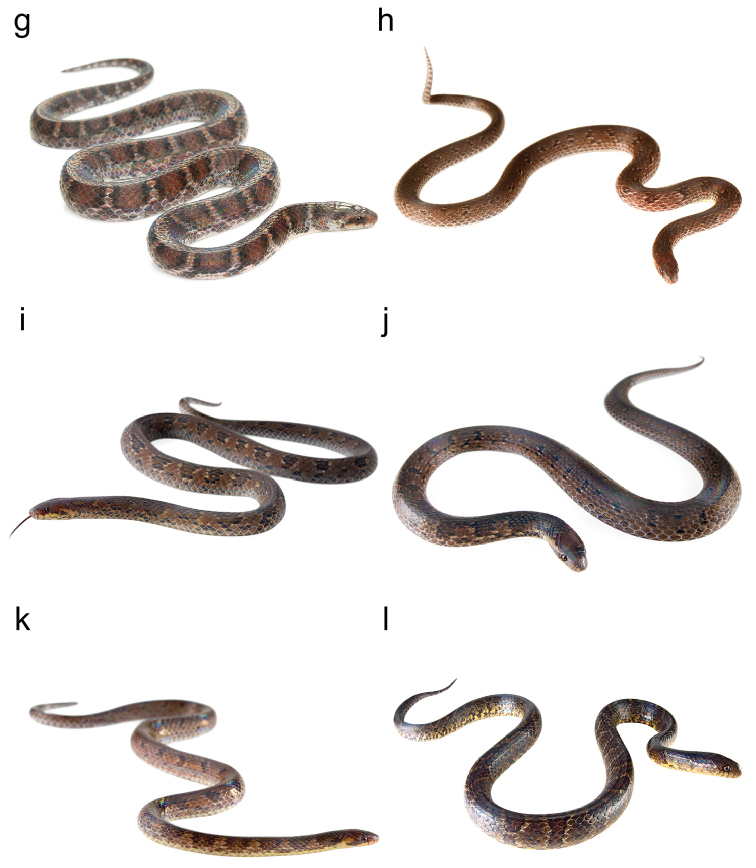
Figure 2. Continued.

**Figure:**
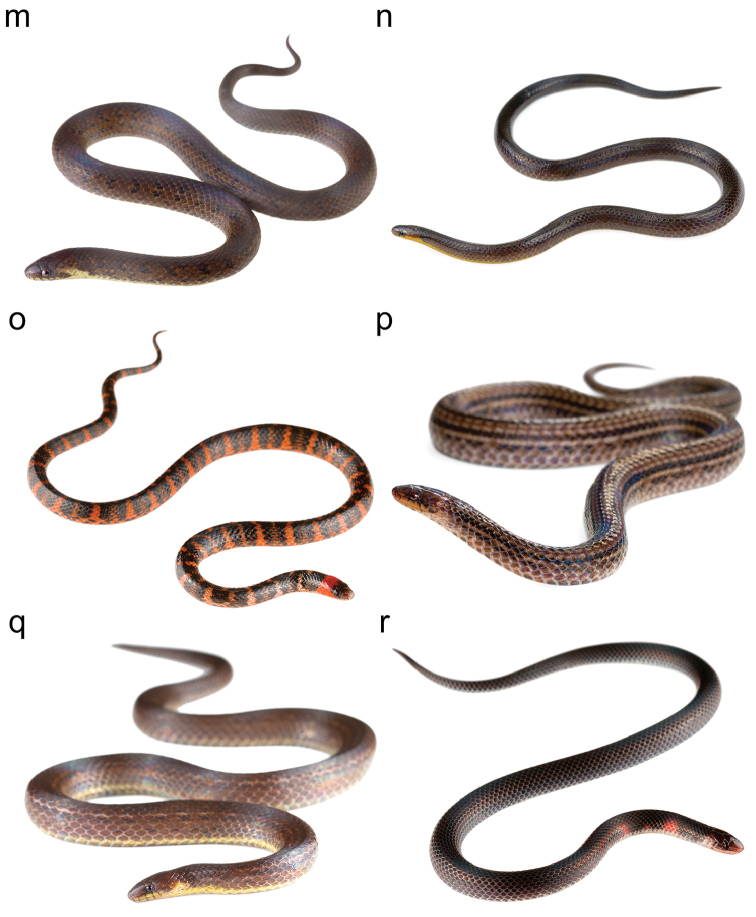
Figure 2. Continued.

**Figure:**
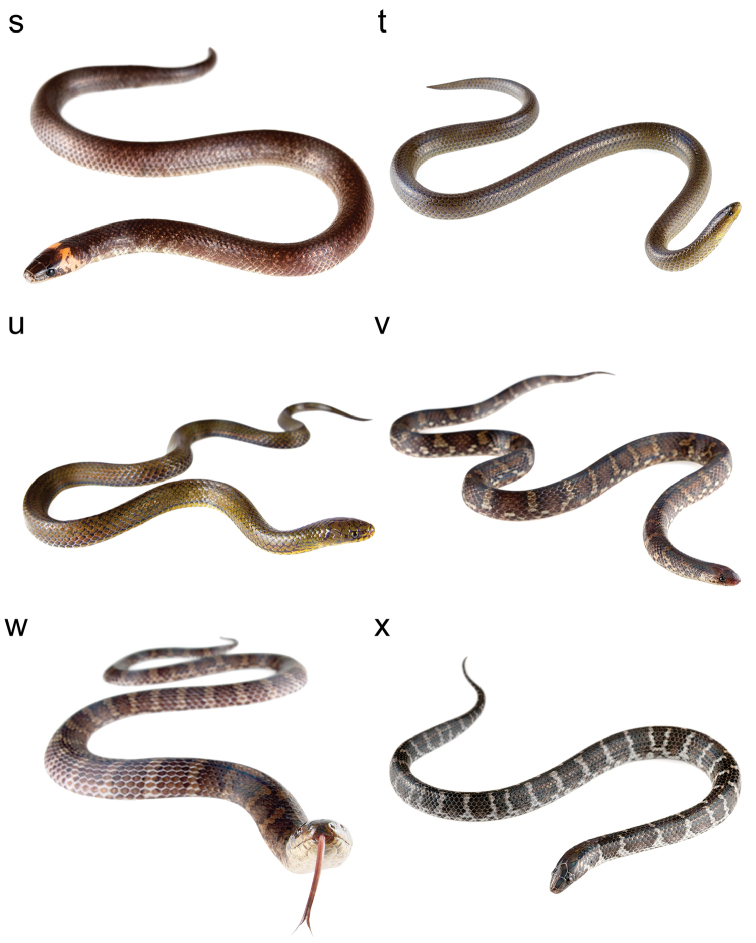
Figure 2. Continued.

**Content.** *Atractus cerberus* sp. n., *Atractus dunni*, *Atractus echidna*, *Atractus esepe* sp. n., *Atractus iridescens*, *Atractus microrhynchus* and *Atractus occidentalis*.

**Table 2.** Morphometric data for members of the *Atractus iridescens* species group. Codes are: V=ventrals; SC=subcaudals; D=dorsal scale rows at midbody; PO=postoculars; SL=supralabials; IL=infralabials; MT=maxillary teeth. Data is derived from Appendix III and from the literature.

Species	V		SC		D	PO	SL	IL	MT
	Males	Females	Males	Females					
*Atractus cerberus*	152–157	–	25–26	–	17	2	7	7	7
*Atractus dunni*	125–136	138–150	26–39	19–26	17	2	6–7	6–8	5–7
*Atractus echidna*	127	–	36	–	15	2	7	7	6
*Atractus esepe*	149	156	41	30	17	2	7	7	5
*Atractus iridescens*	127–150	135–144	33–42	25–37	17	2	6–7	6–7	5–6
*Atractus microrhynchus*	133–150	144–163	32–40	24–29	17	1–2	7	6–7	5–7
*Atractus occidentalis*	129–141	128–149	33–39	20–37	17	2	6–7	6–7	5–7

**Figure 3.**
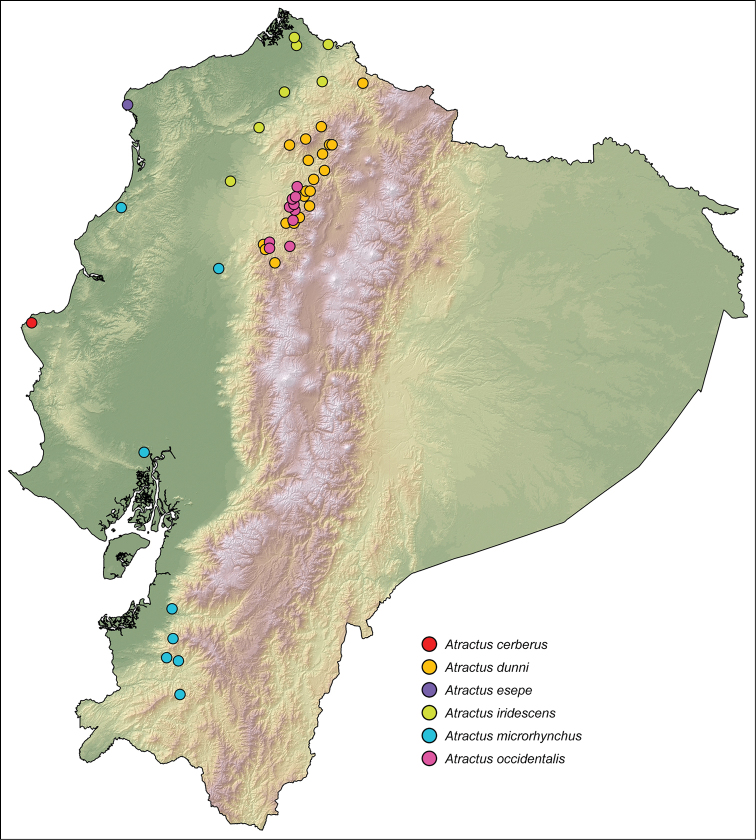
Distribution of Ecuadorian snakes of the *Atractus iridescens* species group. Dots represent known localities.

**Distribution.** Pacific lowlands and western Andean slopes in Ecuador and Colombia (Fig. 3).

**Comment.** Passos et al. (2009a) included *Atractus echidna*, *Atractus iridescens* and *Atractus microrhynchus* in the phenetic *Atractus paucidens* species group. Later, Passos et al. (2012) placed *Atractus microrhynchus* in the *Atractus multicinctus* group based on hemipenial characters. Unlike *Atractus paucidens* or *Atractus multicinctus* (Jan, 1865), however, the former three species have a brownish color pattern (Fig. 2) and also a lower number of ventral scales (Appendix III). These differences, together with the phylogenetic placement of *Atractus iridescens* and *Atractus microrhynchus* support the allocation of these species in the newly formed *Atractus iridescens* group.

### *Atractus roulei* species group

**Diagnosis.** 300–450 mm SVL *Atractus* with olive to grayish brown dorsal ground color lacking dots and stripes, 15/15/15 smooth dorsals (occasionally 17/17/17), generally 6 supralabials (sometimes 5), and 135–161 ventrals (Table 3).

**Table 3.** Morphometric data for members of the *Atractus roulei* species group. Codes are: V=ventrals; SC=subcaudals; D=dorsal scale rows at midbody; PO=postoculars; SL=supralabials; IL=infralabials; MT=maxillary teeth. Data is derived from Appendix III and from the literature.

Species	V		SC		D	PO	SL	IL	MT
	Males	Females	Males	Females					
*Atractus carrioni*	136–151	143–161	25–34	18–32	15	1	6	6	7–10
*Atractus lehmanni*	141–144	148–153	25–29	20–21	15–17	1	5	6	8–11
*Atractus pyroni*	–	143	–	16	15	1	6	5	8
*Atractus roulei*	135–146	143–156	20–27	14–23	15	1	5–6	6–7	9–13

**Content.** *Atractus carrioni*, *Atractus lehmanni*, *Atractus pyroni* sp. n. and *Atractus roulei* (Fig. 1).

**Figure 4.**
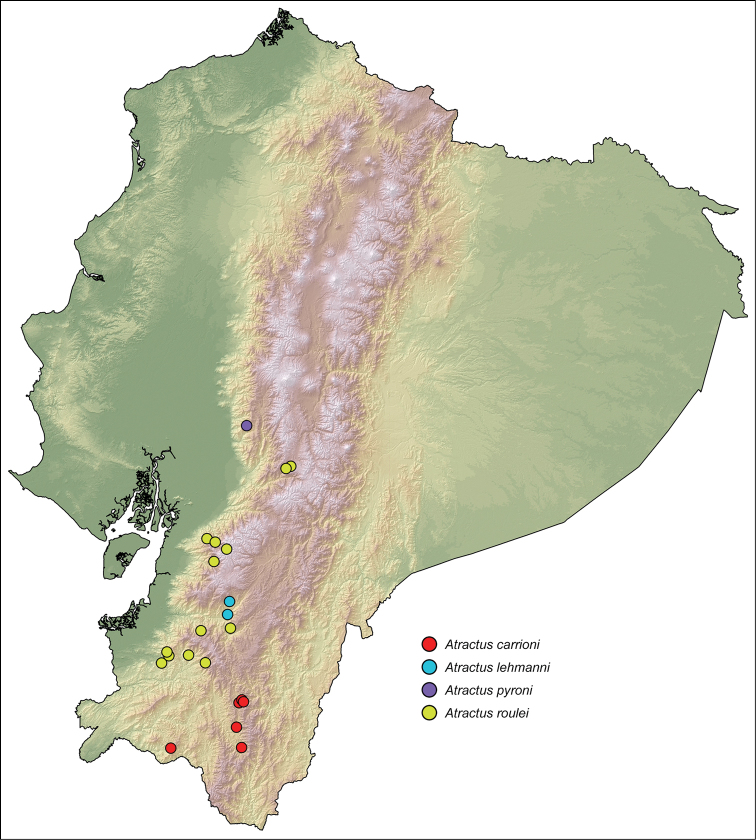
Distribution of Ecuadorian snakes of the *Atractus roulei* species group. Dots represent known localities.

**Distribution.** Western slopes of the Andes and inter-Andean valleys in central and southern Ecuador (Fig. 4).

**Comment.** Passos et al. (2013) created the *Atractus roulei* species group to accommodate *Atractus roulei* and its closest morphological relative *Atractus carrioni*, based mainly on their unusual combination of 15/15/15 dorsals and 6 supralabials. Our examination of new material belonging to these two species, and material belonging to *Atractus pyroni* and *Atractus roulei* (Appendix III), shows that although the majority of specimens have indeed 6 supralabials, some specimens may have 5, compared with most Ecuadorian *Atractus* which have 7 (Appendix III). One specimen of *Atractus roulei* from the type locality (MZUTI 4544; Table 1) lacks a loreal scale, which was long thought (Savage 1960; Passos et al. 2013) to be the main feature separating this species from *Atractus carrioni*. The syntype of *Atractus lehmanni* (MC 33513) revised by Savage (1960) has 17/17/17 dorsal scale rows. Specimens assignable to *Atractus lehmanni* have been found only in the vicinity of the type locality (hoya de Cuenca; see Table 1).

#### Atractus cerberus sp. n.

Taxon classificationAnimaliaSquamataDipsadidae

http://zoobank.org/B93B0063-06B6-462F-8C4B-7559D9459714

##### Proposed standard English name.

Cerberus Groundsnake

##### Proposed standard Spanish name.

Tierrera cancerbera

##### Holotype.

**Figure 5.**
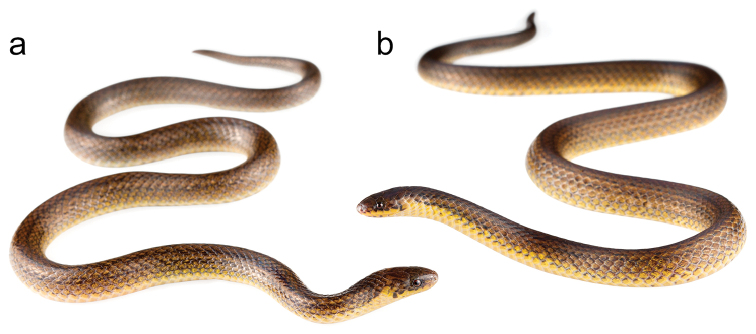
Adult male holotype MZUTI 4330 (**a**) and adult male paratopotype (**b**) of *Atractus cerberus* MZUTI 5108.

MZUTI 4330 (Fig. 5a), adult male collected by José L. Vieira-Fernandes and Alejandro Arteaga on November 06, 2015 at Pacoche, province of Manabí, Ecuador (S1.06664, W80.88123; 280 m).

##### Paratopotype.

MZUTI 5108 (Fig. 5b), adult male collected by Alejandro Arteaga on September 04, 2016.

##### Diagnosis.

*Atractus cerberus* is placed in the genus *Atractus* as diagnosed by Savage (1960), based on phylogenetic evidence (Fig. 1). It is included in the *Atractus iridescens* group due to its brown dorsal ground color (Fig. 5) and its phylogenetic position (Fig. 1). The species is diagnosed based on the following combination of characters: (1) 17/17/17 smooth dorsals; (2) two postoculars; (3) loreal moderate; (4) temporals 1+2; (5) seven supralabials, third and fourth contacting orbit; (6) seven infralabials, first four contacting chinshields (7) seven maxillary teeth; (8) three gular scale rows; (9) three preventrals; (10) 152–157 ventrals; (11) 25–26 subcaudals; (12) dorsal ground color brown with faint black longitudinal bands (Fig. 5); (13) venter light yellow faintly speckled with brownish pigment; (14) 212–309 mm SVL; (15) 23–36 mm TL.

##### Comparisons.

*Atractus cerberus* is included in the *Atractus iridescens* species group and compared to other Pacific lowland congeners that have a brownish ground color (Fig. 2): *Atractus boulengerii*, *Atractus dunni*, *Atractus echidna*, *Atractus esepe* sp. n., *Atractus iridescens*, *Atractus medusa*, *Atractus microrhynchus*, and *Atractus occidentalis*. From *Atractus boulengerii* and *Atractus medusa*, it differs in having a striped pattern as opposed to bold black blotches (Fig. 5). From all others, it differs in having yellow ventral surfaces (as opposed to cream or dingy white) and having more than 150 ventrals in males. Finally, the dorsal pattern of *Atractus cerberus* is less clearly marked than in the majority of the known specimens of the species included in the *Atractus iridescens* group. Instead of having conspicuous spots, blotches or lines, *Atractus cerberus* has a series of feebly visible dashes made of pigment slightly darker than the surrounding ground color.

##### Color pattern.

The dorsal ground color is brown with five feebly visible dark-brown to black longitudinal lines that are not continuous throughout the length of the body but broken into spots along some sections (Fig. 5). Between the dark longitudinal lines on each side of the body, there are fields of lighter pigment that on some sections of the body correspond to lines. The head is darker than the rest of the dorsal surfaces and is marked by a dark, irregular postocular stripe that reaches the corner of the mouth (Fig. 5). The top of the supralabials is tinged with black. The ventral surfaces are yellowish cream with scattered brownish speckling that becomes more concentrated towards the tail, which is almost completely brown. The iris is carmine and the pupil is black.

##### Description of holotype.

Adult male, SVL 212 mm, tail length 23 mm (10.8% SVL); body diameter 6.5 mm; head length 7.9 mm (3.7% SVL); head width 4.8 mm (2.3% SVL); interocular distance 3.1 mm; head slightly distinct from body; snout–orbit distance 2.8 mm; rostral 1.6 mm wide, about one time broader than high; internasals 1.0 mm wide; internasal suture sinistral relative to prefrontal suture; prefrontals 1.7 mm wide; frontal 2.3 mm wide, with a curvilinear triangle shape in dorsal view; parietals 2.1 mm wide, about twice as long as wide; nasal divided; loreal 1.5 mm long, about 2 times longer than high; eye diameter 1.4 mm; pupil round; supraoculars 1.4 mm wide; two postoculars; temporals 1+2, upper posterior temporal elongate, about four times longer than high, and three times as long as first temporal; seven supralabials, 3^rd^–4^th^ contacting orbit; symphisial 1.0 mm wide, about twice as broad as long, separated from chin shields by first pair of infralabials; seven infralabials, 1^st^–4^th^ contacting chin shields; anterior chin shields about three times as long as broad, posterior chin shields absent; three series of gular scales; dorsal scales 17/17/17 rows, smooth without apical pits; preventrals 3; ventrals 157; anal plate single; paired subcaudals 26.

##### Natural history.

The two known specimens of *Atractus cerberus* were found in an isolated patch of deciduous lowland forest surrounded by dry lowland shrubland. MZUTI 4330 was found active on leaf litter at 19h29, in 80% closed canopy secondary forest far from streams. The night was warm and there was drizzle the night before. MZUTI 5108 was found crossing a forest trail close to an open area at 10h00 during a sunny morning after a rainy night.

##### Distribution.

Known only from the type locality, Pacoche, in the Ecuadorian province of Manabí at 280–324 m (Fig. 3). This locality is 3 km airline distance from the shoreline.

##### Etymology.

The specific epithet “*cerberus*” is derived from the name of the Greek monster Kérberos. In Greek mythology, Kérberos is a monstrous multi-headed dog that guards the gates of the underworld, preventing the dead from leaving. Here, we use this word in allusion to the type locality, at the gates of the newly formed “Refinería del Pacífico”, a massive industrial oil-processing plant that can easily be likened to the underworld.

##### Conservation status.

Although *Atractus cerberus* belongs to a poorly studied genus of snakes and is known only from two specimens collected recently in a single locality, we consider this species to be Critically Endagered following B1a,b(iii) IUCN criteria because: i) its extent of occurrence is estimated to be less than 50 km^2^ (i.e. total area of continous semideciduous forest in the Refugio de Vida Silvestre Pacoche); ii) it has not been detected in any other locality in the province of Manabí despite numerous surveys (Almendáriz and Carr 2007, Cisneros-Heredia 2004, MECN et al. 2013); and iii) and its habitat is severely fragmented, isolated from other such habitats and declining in extent and quality due to deforestation.

#### Atractus esepe sp. n.

Taxon classificationAnimaliaSquamataDipsadidae

http://zoobank.org/F58E89A5-D398-4703-8098-7474CD6B3E6D

##### Proposed standard English name.

Indistinct Groundsnake

##### Proposed standard Spanish name.

Tierrera indistinta

##### Holotype.

**Figure 6.**
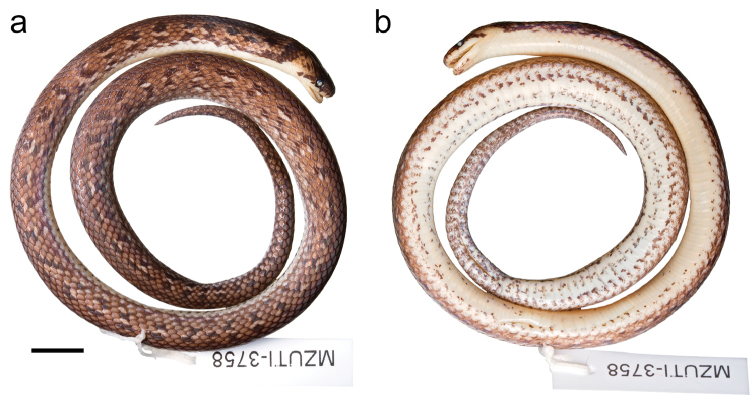
Adult male holotype of *Atractus esepe* MZUTI 3758 in dorsal (**a**) and ventral (**b**) view. Scale = 1 cm.

MZUTI 3758 (Fig. 6), adult male collected by Alejandro Arteaga on September 12, 2014 at Caimito, Esmeraldas Province, Ecuador (N0.69620, W80.090472; 102 m).

##### Paratopotype.

MZUTI 3759, adult female collected by Jaime Culebras.

##### Diagnosis.

*Atractus esepe* is placed in the genus *Atractus* as diagnosed by Savage (1960), based on phylogenetic evidence (Fig. 1). It is included in the *Atractus iridescens* group due to its brown dorsal ground color and its phylogenetic position (Figs 1, 6). The species is diagnosed based on the following combination of characters: (1) 17/17/17 smooth dorsals; (2) two postoculars; (3) loreal long; (4) temporals 1+2; (5) seven supralabials, third and fourth contacting orbit; (6) seven infralabials, first four contacting chinshields (7) seven maxillary teeth; (8) 2–3 gular scale rows; (9) 2–3 preventrals; (10) 149 ventrals in the male holotype, 156 in the female paratype; (11) 41 subcaudals in the male holotype, 30 in the female paratype; (12) dorsal ground color brown with a pattern of complete (MZUTI 3759) or broken (MZUTI 3758) (Fig. 6a) dark lines running parallel along each side of the body and separated from each other by a cream line, but rendering the appearance of a row of dorso-lateral blotches in the broken pattern (MZUTI 3758); (13) venter cream faintly speckled with brownish pigment (Fig. 6b); (14) 232–241 mm SVL; (15) 34–53 mm TL.

##### Comparisons.

*Atractus esepe* is included in the *Atractus iridescens* species group and compared to other Pacific lowland congeners who have a brownish ground color (Figs 2, 5): *Atractus boulengerii*, *Atractus cerberus*, *Atractus dunni*, *Atractus echidna*, *Atractus iridescens*, *Atractus medusa*, *Atractus microrhynchus*, and *Atractus occidentalis*. From these, *Atractus microrhynchus* and *Atractus occidentalis* have striped pattern and cream ventral surfaces similar to that of *Atractus esepe*, but they occur parapatrically (Fig. 3) and can be distinguished from *Atractus esepe* by a genetic divergence of 5.3–5.7% in a 506 bp long fragment of the mitochondrial Cytb gene and by having a greater number of subcaudal scales in males (Table 2). Furthermore, adult specimens of *Atractus microrhynchus* have light brown dorsal surfaces instead of dark brown, and their pattern can be better described as a series of blotches rather than broken longitudinal lines. Specimens of both *Atractus esepe* and *Atractus occidentalis* have a pattern of longitudinal lines, but *Atractus esepe* has a greater number of ventral plus caudal scales than *Atractus occidentalis* (more than 180 in *Atractus esepe*) (Table 2).

##### Color pattern.

The dorsal ground color is dark brown with either six longitudinal black lines separated by lighter areas or a pattern of dark brown longitudinally arranged spots that correspond to the longitudinal lines. On each side, the line or series of dark spots along the 2^nd^ and 3^rd^ dorsal scale row is feebly visible, but the other lines or spots are conspicuous. The dorsal surface of the head is dark brown and there is a clearly marked dark postocular stripe running from behind the eye to the edge of the mouth (Fig. 6). The ventral surfaces are dingy white, finely speckled with brown pigment that becomes more concentrated towards the tail. The iris is carmine and the pupil is black.

##### Description of holotype.

Adult male, SVL 232 mm, tail length 53 mm (22.8% SVL); body diameter 7.0 mm; head length 7.9 mm (3.4% SVL); head width 4.8 mm (2.2% SVL); interocular distance 3.4 mm; head slightly distinct from body; snout–orbit distance 3.3 mm; rostral 1.8 mm wide, about one time broader than high; internasals 0.9 mm wide; internasal suture sinistral relative to prefrontal suture; prefrontals 1.9 mm wide; frontal 2.2 mm wide, with a curvilinear triangle shape in dorsal view; parietals 2.1 mm wide, about twice as long as wide; nasal divided; loreal 2.5 mm long, about 3 times longer than high; eye diameter 1.5 mm; pupil round; supraoculars 1.2 mm wide; two postoculars; temporals 1+2, upper posterior temporal elongate, about four times longer than high, and three times as long as first temporal; seven supralabials, 3^rd^–4^th^ contacting orbit; symphisial 0.8 mm wide, separated from chin shields by first pair of infralabials; seven infralabials, 1^st^–4^th^ contacting chin shields; anterior chin shields about three times as long as broad, posterior chin shields absent; three series of gular scales; dorsal scales 17/17/17 rows, smooth without apical pits; preventrals 3; ventrals 149; anal plate single; paired subcaudals 41.

##### Natural history.

The two known specimens of *Atractus esepe* were found actively foraging among soil and roots in secondary evergreen lowland forest at least 400 m from the nearest natural body of water. They were found by night at 20h00 after a warm, sunny day.

##### Distribution.

Known only from the type locality, Caimito, in the Ecuadorian province of Esmeraldas at 102 m (Fig. 3). This locality is 1.3 km airline distance from the shoreline.

##### Etymology.

The specific epithet *esepe* is derived from the Spanish pronunciation of “sp.”, which is the abbreviation for the Latin word *species*. Here, we use this word in allusion to how the majority of Ecuadorian researchers refer to *Atractus* specimens found in the field.

##### Conservation status.

We consider *Atractus esepe* to be Data Deficient following IUCN criteria because it is known only from its type locality but its occurrence in the biogeographic Choco suggests that it might as well be present in other localities. The Chocoan forests of Caimito do not appear to be isolated from other similar habitat by geographical or ecological barriers. Therefore, we consider there is inadequate information to make a direct, or indirect, assessment of its extinction risk based on its scarce distribution data.

#### Atractus pyroni sp. n.

Taxon classificationAnimaliaSquamataDipsadidae

http://zoobank.org/36145E29-02B6-4C66-A097-44EFC1BC3A92

##### Proposed standard English name.

Pyron’s Groundsnake

##### Proposed standard Spanish name.

Tierrera de Pyron

##### Holotype.

**Figure 7.**
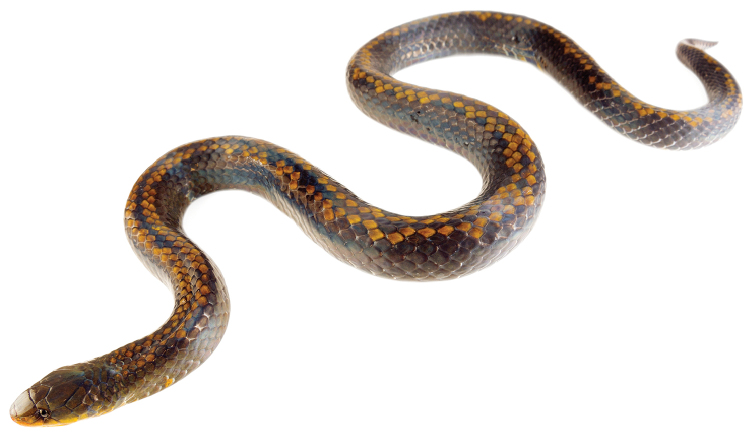
Adult female holotype of *Atractus pyroni*. MZUTI 5107. Scale = 1 cm.

MZUTI 5107 (Fig. 7), adult male collected by José L. Vieira-Fernandes and Carlos Durán on May 23, 2016 between Balzapamba and Bilován, province of Bolívar, Ecuador (S1.83601, W79.13322; 2026 m).

##### Diagnosis.

*Atractus pyroni* is placed in the genus *Atractus* as diagnosed by Savage (1960), based on phylogenetic (Fig. 1) and morphological (Table 3) evidence. It is included in the *Atractus roulei* group due to its 15/15/15 dorsal scale rows and its phylogenetic position (Fig. 1). The species is diagnosed based on the following combination of characters: (1) 15/15/15 smooth dorsals; (2) one postocular; (3) loreal long; (4) temporals 1+2; (5) six supralabials, third and fourth contacting orbit; (6) five infralabials, first four contacting chinshields (7) eight maxillary teeth; (8) 3 gular scale rows; (9) 2 preventrals; (10) 143 ventrals; (11) 16 subcaudals; (12) dorsal ground color dark brown with a series of light golden brown paravertebral scales running along the entire dorsum (Fig. 7); (13) venter dark brown with scattered scales of a lighter color; (14) 443 mm SVL; (15) 34 mm TL.

##### Comparisons.

*Atractus pyroni* is compared to members of the *Atractus roulei* species group: *Atractus carrioni*, *Atractus lehmanni*, and *Atractus roulei* (Fig. 2). From *Atractus carrioni*, it differs by having a loreal. From *Atractus lehmanni* and *Atractus roulei*, it differs in size and color pattern. *Atractus pyroni* is 443 mm in SVL; whereas *Atractus lehmanni* is 262–321 in SVL, and *Atractus roulei* is 230–396. Both *Atractus lehmanni* and *Atractus roulei* have uniform dorsal ground color, whereas *Atractus pyroni* has a distinct dorsal bicolored pattern (Fig. 7). Finally, in life, *Atractus pyroni* is darker than the remaining members of the *Atractus roulei* species group and has a ventral pattern that, instead of having fine speckles, has conspicuous scattered blotches of a contrasting color.

##### Color pattern.

The dorsal ground color is blackish with a dark vertebral (mid-dorsal) scale row flanked by a dark yellow scale row on either side (the 7^th^ dorsal scale row), irregularly adjoined by one to few additional yellow scales on the 6^th^ dorsal scale row, rendering an appearance of an irregularly edged mid-dorsal striped pattern (Fig. 7). The dorsal and lateral surfaces of the head are dark grayish brown and the labials are dark mustard yellow. All ventral surfaces are glossy grayish black except for the throat and some scattered blotches, which are dark mustard yellow.

##### Description of holotype.

Adult female, SVL 443 mm, tail length 34 mm (7.7% SVL); body diameter 11.6 mm; head length 14.4 mm (3.3% SVL); head width 9.8 mm (2.2% SVL); interocular distance 5.1 mm; head slightly distinct from body; snout–orbit distance 5.7 mm; rostral 2.8 mm wide, about two times broader than high; internasals 1.5 mm wide; internasal suture sinistral relative to prefrontal suture; prefrontals 2.8 mm wide; frontal 3.5 mm wide, with a curvilinear triangle shape in dorsal view; parietals 4.0 mm wide, about twice as long as wide; nasal divided; loreal 3.7 mm long, about 3 times longer than high; eye diameter 1.8 mm; pupil round; supraoculars 2.1 mm wide; one postocular; temporals 1+2, upper posterior temporal elongate, about five times longer than high, and twice as long as first temporal; six supralabials, 3^rd^–4^th^ contacting orbit; symphisial 2.4 mm wide, separated from chin shields by first pair of infralabials; five infralabials, 1^st^–4^th^ contacting chin shields; anterior chin shields about three times as long as broad, posterior chin shields absent; three series of gular scales; dorsal scales 15/15/15 rows, smooth without apical pits; preventrals 2; ventrals 143; anal plate single; paired subcaudals 16.

##### Natural history.

The only known specimen of *Atractus pyroni* was found dead on a dirt road surrounded by silvopastures and remnants of native montane cloudforest.

##### Distribution.

Known only from the type locality, between Balzapamba and Bilován, in the Ecuadorian province of Bolívar at 2026 m (Fig. 7).

##### Etymology.

Named after R. Alexander Pyron, one of the most prolific contemporary herpetologists, in recognition of his invaluable contribution to systematics and evolution of the world’s reptiles.

##### Conservation status.

We consider *Atractus pyroni* to be to be Data Deficient following IUCN because there is inadequate information to make a direct, or indirect, assessment of its extinction risk based on its scarce distribution data.

## Discussion

Species relationships and taxonomy in the colubrid snake genus *Atractus* are still far from being resolved, and many infrageneric groups are either non-monophyletic, or poorly supported and weakly placed, which may reflect inadequate sampling of taxa (only 30 out of 140 species are included) or characters (only 1 locus is used). No monophyly was found for the groups defined by Savage (1960), which, until further phylogenetic evidence is accumulated or unambiguous diagnostic characters are defined, should not be used.

From the five members of the *Atractus paucidens* species groups of Passos et al. (2009a) that were sampled in our phylogeny, only *Atractus paucidens*, *Atractus savagei*, and *Atractus typhon* cluster together. *Atractus microrhynchus* and *Atractus iridescens* belong to another lineage, which is here named the *Atractus iridescens* species group. This group includes the aforementioned two species plus *Atractus cerberus*, *Atractus dunni*, *Atractus echidna*, *Atractus esepe*, and *Atractus occidentalis*. From the species included in this group, we expand the known distribution of all their members (Fig. 3). However, we do not include the specimens ANSP 18114 nor ANSP 26316, from the vicinity of Huigra and identified as *Atractus occidentalis* by Savage (1960), because their description disagrees with the observed morphological variation reported for *Atractus occidentalis* in this work. Upon a visit to Huigra, a dry valley dominated by xeric vegetation and rocky outcrops, it became clear to us that it is unlikely for a species like *Atractus occidentalis*, which is found in evergreen lower-montane forests (Arteaga et al. 2013), to occur in an isolated dry habitat type ca. 250 km airline distance south of the type locality.

We also re-delimit the *Atractus roulei* species group of Passos et al. (2013) to include *Atractus carrioni*, *Atractus lehmanni*, *Atractus roulei* and *Atractus pyroni*. We expand the known distribution of *Atractus roulei* (Fig. 4), but do not include specimen AMNH 17492 from San José de Chimbo (Savage 1960) in the map because this specimen might actually be *Atractus pyroni* given the morphological similarities between the two species and the geographical proximity to the type locality of *Atractus pyroni*. Reports of *Atractus lehmanni* from Colombia (Passos et al. 2009b) are likely misidentifications since *Atractus lehmanni* has not been registered in Ecuador outside the type locality.

To further clarify the landscape of *Atractus* taxonomy in Ecuador, we analyze the presence of *Atractus medusa*, *Atractus melas*, *Atractus typhon*, *Atractus badius*, and *Atractus bocourti* in the country. Cisneros-Heredia and Romero (2015) presented the first country record of *Atractus medusa* in Ecuador (specimen DFCH-USFQ 191.101109 at Universidad San Francisco de Quito), based on similarities in scalation and coloration between that specimen and the holotype of *Atractus medusa*, form Gorgona island, Colombia. Certainly, the characters of scalation of the Ecuadorian specimen fit the diagnosis of *Atractus medusa*. However, they fit just as well the diagnosis of *Atractus iridescens* provided by Passos et al. (2009a), with the difference that the dorsal pattern of the Ecuadorian specimen resembles more the *Atractus iridescens* specimen, ICN 10902, pictured in Passos et al. (2009a). The dark brown ground color (as opposed to light cream), the light bordered brown blotches (as opposed to solid black blotches), and the absence of a black nape band are all characteristics shared by DFCH-USFQ 191.101109 and the other nine specimens of *Atractus iridescens* presented in Appendix III, with ICN 10902 of Passos et al. (2009a). Therefore, we consider that DFCH-USFQ 191.101109 actually represents the first country record of *Atractus iridescens* for Ecuador. Based on this new information and re-examination of museum material, we report on 9 additional specimens (Table 1) that expand the current known distribution of this species. Cisneros-Heredia and Romero (2015) suggest that a photographic record of Atractus cf. melas from the Bilsa Biological Station, province of Esmeraldas, northwestern Ecuador (Ortega-Andrade et al. 2010) corresponds to *Atractus multicinctus*. The specimen differs from other material assigned to *Atractus multicinctus* in having whitish rings as opposed to red rings throughout the body (Fig. 2). Although photographic vouchers of *Atractus typhon* have been presented in MECN et al. (2013), we report on the first museum vouchers of the species in Ecuador (Table 1).

Finally, although Hoogmoed (1980) restricted the type locality of *Atractus badius* and pointed out that the upper Amazon basin specimens were misidentifications, the species has remained in Ecuadorian faunal lists (Torres-Carvajal et al. 2016), even after Schargel et al. (2013) made compelling cases to exclude this species from the upper Amazon Basin. Other snake, *Atractus bocourti* was included in the herpetofauna of Ecuador by Pérez-Santos and Moreno (1991) without pointing out to any museum voucher. These authors stated that although they have no information about the distribution of the species in Ecuador, its distribution in Colombia would suggest that it also occurs in Ecuador. Since there is no evidence that neither *Atractus badius* nor *Atractus bocourti* occur in Ecuador, we remove them from this country’s herpetofauna.

Our analysis of new *Atractus* material supports the evolutionary phylogenetic distinctiveness of at least 22 of the total taxa currently recognized to occur in Ecuador. To include the remaining taxa in future phylogenetic analyses will certainly help resolve species relationships and taxonomic arrangements of cis-Andean Ecuadorian *Atractus*, since the five species that were not included in the phylogeny occur in the Amazonian slopes of the Andes. However, besides including more taxa in future phylogenetic analyses, we feel that a more adequate sampling of molecular markers is needed to overcome the difficulties that mitochondrial-based phylogenies have to capture higher-lever evolutionary relationships. Certainly, future studies can benefit from a phylogeny based on both a nuclear and a mitochondrial dataset.

With these changes, the species number reported in Ecuador increases to 27: *Atractus carrioni* (Parker, 1930), *Atractus cerberus*, *Atractus collaris* (Peracca, 1897), *Atractus duboisi* (Boulenger, 1880), *Atractus dunni* (Savage, 1955), *Atractus ecuadorensis* (Savage, 1955), *Atractus elaps* (Günther, 1858), *Atractus esepe*, *Atractus gaigeae* (Savage, 1955), *Atractus gigas* (Myers and Schargel, 2006), *Atractus iridescens* (Peracca, 1860), *Atractus lehmanni* (Boettger, 1898), *Atractus major* (Boulenger, 1894), *Atractus microrhynchus* (Cope, 1868), *Atractus modestus* (Boulenger, 1894), *Atractus multicinctus* (Jan, 1865), *Atractus occidentalis* (Savage, 1955), *Atractus occipitoalbus* (Jan, 1862), *Atractus orcesi* (Savage, 1955), *Atractus paucidens* (Despax, 1910), *Atractus pyroni*, *Atractus resplendens* (Werner, 1901), *Atractus roulei* (Despax, 1910), *Atractus savage* (Salazar-Valenzuela et al., 2014), *Atractus snethlageae* (Da Cunha & Do Nascimento, 1983), *Atractus touzeti* (Schargel et al., 2013) and *Atractus typhon* (Passos et al., 2009).

We hope that the novel genetic and morphological data provided herein will promote future researchers to examine species boundaries in *Atractus*, as additional work clearly is waiting.

## Author contributions

Conceived and designed the work: AA. Performed the analyses: AA NP. Gathered morphological data: KB JHV DFCH CRP JLVF AA. Analyzed the data: AA KM DFCH JMG. Contributed reagents/materials/analysis tools: JMG NP. Wrote the paper: AA KM JHV DFCH NP CRP JLVF JMG.

## Supplementary Material

XML Treatment for Atractus cerberus

XML Treatment for Atractus esepe

XML Treatment for Atractus pyroni

## Appendix I

**Table:** GenBank accession numbers for loci and terminals of taxa and outgroups sampled in this study. Novel sequence data produced in this study are marked with an asterisk (*).

Species	Voucher	16S	CYTB	ND4
*Atractus albuquerquei*	–	GQ457726	JQ598918	–
*Atractus badius*	–	AF158485	–	–
*Atractus carrioni*	MZUTI 4195	KY610046*	–	KY610094*
*Atractus cerberus*	MZUTI 4330	KY610047*	KY610073*	KY610095*
*Atractus duboisi*	MZUTI 62	KT944041	–	KT944059
*Atractus dunni*	MZUTI 2189	KY610048*	–	KY610096*
*Atractus dunni*	MZUTI 3031	KY610049*	–	KY610097*
*Atractus dunni*	MZUTI 4318	KY610050*	KY610074*	KY610098*
*Atractus dunni*	MZUTI 4319	KY610051*	KY610075*	KY610099*
*Atractus ecuadorensis*	DHMECN 5105	–	–	KY610100*
*Atractus elaps*	DHMECN 10179	KY610052*	KY610076*	KY610101*
*Atractus elaps*	KU 214837	–	EF078536	EF078584
*Atractus esepe*	MZUTI 3758	KY610053*	KT944052	KY610102*
*Atractus esepe*	MZUTI 3759	KT944039	KT944051	KT944058
*Atractus flammigerus*	MNHN 1997.2145	AF158471	–	–
*Atractus gigas*	MZUTI 3286	KT944043	KT944053	KT944061
*Atractus iridescens*	DHMECN 9633	KY610054*	KY610077*	–
*Atractus iridescens*	MZUTI 3548	KY610055*	KY610078*	–
*Atractus iridescens*	MZUTI 3680	KY610056*	KY610079*	–
*Atractus iridescens*	MZUTI 4178	KT944040	KY610080*	–
*Atractus iridescens*	MZUTI 4697	KY610057*	KY610081*	–
*Atractus lehmanni*	DHMECN 7644	KY610058*	KY610082*	KY610103*
*Atractus major*	ANF 1545	KT944045	–	KY610104*
*Atractus major*	DHMECN 8343	KY610059*	–	KY610105*
*Atractus microrhynchus*	MZUTI 5109	KY610060*	KY610083*	KY610106*
*Atractus microrhynchus*	MZUTI 4122	KT944037	KT944049	KT944056
*Atractus modestus*	MZUTI 4760	KY610061*	KY610084*	KY610107*
*Atractus multicinctus*	MZUTI 5106	KY610062*	KY610085*	KY610108*
*Atractus occidentalis*	MZUTI 1385	KY610063*	KY610086*	KY610109*
*Atractus occidentalis*	MZUTI 2649	KY610064*	KY610087*	KY610110*
*Atractus occidentalis*	MZUTI 2650	KT944038	KT944050	KT944057
*Atractus occidentalis*	MZUTI 3323	KY610065*	KY610088*	KY610111*
*Atractus paucidens*	MZUTI 5102	KY610066*	–	KY610112*
*Atractus paucidens*	MZUTI 5104	–	–	KY610113*
*Atractus paucidens*	MZUTI 5105	KY610067*	–	KY610114*
*Atractus pyroni*	MZUTI 5107	KY610068*	KY610089*	KY610115*
*Atractus resplendens*	MZUTI 3996	KT944042	KT944055	KT944060
*Atractus roulei*	MZUTI 4503	–	KY610090*	KY610116*
*Atractus roulei*	MZUTI 4544	KY610069*	KY610091*	KY610117*
*Atractus savagei*	MZUTI 4916	KY610070*	KY610092*	KY610118*
*Atractus schach*	–	AF158486	–	–
*Atractus touzeti*	ANF 2390	KY610071*	KY610093*	KY610119*
*Atractus trihedrurus*	–	GQ457727	JQ598919	–
*Atractus typhon*	DHMECN 9632	KY610072*	–	KY610120*
*Atractus typhon*	MZUTI 3284	KT944044	KT944054	KT944062
*Atractus wagleri*	MHUA 14368	–	GQ334480	GQ334581
*Atractus zebrinus*	–	JQ598861	–	–
*Atractus zidocki*	MNHN 1997.2046	AF158487	–	–
Outgroups				
*Geophis godmani*	–	JQ598877	JQ598932	–
*Sibon nebulatus*	MVZ 233298	EU728583	EU728583	EU728583

## Appendix II

**Table:** List of PCR and sequencing primers and their respective PCR conditions (denaturation, annealing, extension and number of corresponding cycles) used in this study. All PCR protocols included an initial 3-min step at 94 °C and a final extension of 10 min at 72 °C.

Locus	Primer name	Sequence (5’-3’)	Reference	PCR profile:
16S	16Sar-L	CGCCTGTTTATCAAAAACAT	Palumbi et al. (1991)	94 °C (45 sec), 53 or 56 °C (45 sec), 72 °C (1 min) [x25-30]
	16Sbr-H-R	CCGGTCTGAACTCAGATCACGT		
Cytb	L14910	GACCTGTGATMTGAAAACCAYCGTTGT	Burbrink et al. (2000)	94 °C (1 min), 58 °C (1 min), 72 °C (2 min) [x30-36]
	H16064	CTTTGGTTTACAAGAACAATGCTTTA		
ND4	ND4	CACCTATGACTACCAAAAGCTCATGTAGAAGC	Arévalo et al. (1994)	94 °C (25 sec), 58 or 60 °C (1 min), 72 °C (2 min) [x25-30]
	Leu	CATTACTTTTACTTGGATTTGCACCA		

## Appendix III

**Table:** Morphometric data and sex for specimens of *Atractus* species examined. Codes are: V=ventrals; SC=subcaudals; D1–3=dorsal scale rows at neck, midbody, and vent; PO=postoculars; SL=supralabials; IL=infralabials; MT=maxillary teeth; SVL=snout-vent length (mm); TL=tail length (mm); M=Male, F=Female.

Species	Voucher	V	SC	D1	D2	D3	PO	SL	IL	MT	SVL	TL	Sex
*Atractus carrioni*	DHMECN 4697	144	32	15	15	15	1	6	6	7	361	59	F
*Atractus carrioni*	DHMECN 76	157	23	15	15	15	1	6	6	8	333	39	F
*Atractus carrioni*	DHMECN 7668	149	28	15	15	15	1	6	6	7	354	58	M
*Atractus carrioni*	MZUTI 4195	144	31	15	15	15	1	6	6	8	371	53	M
*Atractus cerberus*	MZUTI 5108	152	25	17	17	17	2	7	7	7	309	36	M
*Atractus cerberus*	MZUTI 4330	157	26	17	17	17	2	7	7	7	212	23	M
*Atractus duboisi*	MHNG 2457.093	166	22	15	15	15	2	7	6	6	455	34	F
*Atractus duboisi*	MNHN 0.6147	164	17	15	15	15	2	8	7	–	131	11	F
*Atractus dunni*	DHMECN 12769	141	36	17	17	17	2	6	7	7	279	39	–
*Atractus dunni*	DHMECN 2215	144	24	17	17	17	2	7	7	6	278	35	F
*Atractus dunni*	DHMECN 3527	141	24	17	17	17	2	6	6	6	352	48	F
*Atractus dunni*	DHMECN 3900	143	21	17	17	17	2	6	6	–	101	19	–
*Atractus dunni*	DHMECN 4159	129	35	17	17	17	2	5	6	6	266	65	–
*Atractus dunni*	EPN 3127	–	–	–	–	–	–	–	–	–	355	46	F
*Atractus dunni*	EPN 3128	–	–	–	–	–	–	–	–	–	295	63	M
*Atractus dunni*	FHGO 375	128	36	17	17	17	2	7	7	6	219	48	M
*Atractus dunni*	FHGO 376	143	26	17	17	17	2	7	7	5	278	33	F
*Atractus dunni*	FHGO 379	132	35	17	17	17	2	7	7	6	297	61	M
*Atractus dunni*	FHGO 91	125	35	17	17	17	2	7	7	6	231	52	M
*Atractus dunni*	MHNG 2441.043	145	20	17	17	17	2	7	7	6	205	22	F
*Atractus dunni*	MHNG 2457.091	129	34	17	17	17	2	7	6	5	197	39	M
*Atractus dunni*	MHNG 2464.03	136	39	16	17	17	2	7	6	5	114	22	M
*Atractus dunni*	MZUTI 2189	134	29	17	17	17	2	7	7	6	189	28	M
*Atractus dunni*	MZUTI 3031	139	24	17	17	17	2	7	7	5	329	36	F
*Atractus dunni*	MZUTI 4097	149	21	17	17	17	2	7	7	6	152	17	–
*Atractus dunni*	MZUTI 4098	130	37	17	17	17	2	7	7	6	126	19	–
*Atractus dunni*	MZUTI 4099	140	25	17	17	17	2	7	7	–	118	15	F
*Atractus dunni*	MZUTI 4100	138	24	17	17	17	2	7	7	–	335	36	F
*Atractus dunni*	MZUTI 4318	136	34	17	18	17	2	7	7	6	242	53	M
*Atractus dunni*	MZUTI 4319	129	35	15	17	17	2	7	7	5	242	53	M
*Atractus esepe*	MZUTI 3758	149	41	17	17	17	2	7	7	5	232	53	M
*Atractus esepe*	MZUTI 3759	156	30	17	17	17	2	7	7	5	241	34	F
*Atractus gaigeae*	MHNG 2397.044	136	34	17	17	17	2	7	7	5	129	23	M
*Atractus gigas*	MHNG 2250.035	168	34	19	17	17	2	6	6	3	272	40	F
*Atractus gigas*	MHNG 2441.02	177	31	17	17	17	2	6	6	5	1060	116	F
*Atractus iridescens*	DHMECN 2932	138	28	17	17	17	2	6	7	6	252	36	F
*Atractus iridescens*	DHMECN 5663	141	32	17	17	17	2	6	6	6	272	46	F
*Atractus iridescens*	DHMECN 9633	129	42	16	17	17	2	6	6	6	219	62	M
*Atractus iridescens*	FHGO 10443	139	32	17	17	17	2	7	7	5	204	32	F
*Atractus iridescens*	MZUTI 3548	131	34	17	17	17	2	7	7	6	200	44	M
*Atractus iridescens*	MZUTI 3680	140	40	17	17	17	2	7	7	6	210	46	M
*Atractus iridescens*	MZUTI 4178	148		17	17	17	2	7		5	211	37	M
*Atractus iridescens*	MZUTI 4697	127	38	17	17	17	2	7	7	5	209	46	M
*Atractus lehmanni*	DHMECN 7644	144	29	15	15	15	1	5	6	11	300	35	M
*Atractus lehmanni*	DHMECN 7645	144	23	15	15	15	1	5	7	10	321	42	–
*Atractus major*	MNHN 0.6149	174	35	17	17	17	2	7	7	6	586	86	F
*Atractus microrhynchus*	DHMECN 2586	144	39	17	17	17	1	7	6	6	239	45	M
*Atractus microrhynchus*	FHGO 897	149	37	17	17	17	2	7	7	7	239	51	M
*Atractus microrhynchus*	MHNG 2307.017	133	34	18	17	17	2	7	6	5	269	55	M
*Atractus microrhynchus*	MHNG 2397.019	144	25	17	17	17	2	7	7	6	300	–	F
*Atractus microrhynchus*	MHNG 2397.02	147	26	17	17	17	2	7	6	5	225	28	F
*Atractus microrhynchus*	MHNG 2397.021	144	24	17	17	17	2	7	6	5	217	28	F
*Atractus microrhynchus*	MHNG 2459.052	137	36	17	17	17	2	7	6	5	239	53	M
*Atractus microrhynchus*	MZUTI 4122	163	29	17	17	17	2	7	7	7	222	27	F
*Atractus microrhynchus*	QCAZ 1219	147	40	17	17	17	2	7	7	7	178	37	M
*Atractus microrhynchus*	USNM 285473	152	26	17	17	17	2	7	7	–	335	45	F
*Atractus microrhynchus*	USNM 285474	163	28	17	17	17	2	7	7	–	212	21	F
*Atractus modestus*	DHMECN 3859		45	17	17	17	2	7	6	–	344	41	–
*Atractus modestus*	FHGO 2936	165	41	17	17	17	2	7	7	5	110	20	M
*Atractus modestus*	FHGO 44	186	27	17	17	17	2	7	7	6	294	38	F
*Atractus modestus*	MHNG 2397.041	146	21	15	15	15	2	7	6	6	200	23	M
*Atractus modestus*	MZUTI 4760	147	42	17	17	17	2	6	7	5	273	59	M
*Atractus occidentalis*	FHGO 385	128	37	17	17	17	2	7	7	7	188	40	F
*Atractus occidentalis*	MHNG 2252.079	145	20	17	17	17	2	6	7	5	262	25	F
*Atractus occidentalis*	MHNG 2307.068	141	35	17	17	17	2	6	7	5	272	55	M
*Atractus occidentalis*	MHNG 2397.028	137	38	17	17	17	2	6	7	5	117	21	M
*Atractus occidentalis*	MHNG 2411.085	138	35	17	17	17	2	7	7	5	253	55	M
*Atractus occidentalis*	MHNG 2411.086	129	33	17	17	17	2	7	6	5	122	23	M
*Atractus occidentalis*	MHNG 2441.044	134	37	17	17	17	2	7	7	–	274	68	M
*Atractus occidentalis*	MZUTI 2649	134	36	17	17	16	2	7	7	6	223	35	F
*Atractus occidentalis*	MZUTI 2650	149	24	17	17	17	2	7	7	–	191	21	F
*Atractus occidentalis*	MZUTI 3323	134	39	17	17	17	2	7	7	7	332	67	M
*Atractus paucidens*	DHMECN 11980	171	43	17	17	17	2	7	7	7	290	50	M
*Atractus paucidens*	DHMECN 3975	163	43	17	17	17	2	–	7	7	249	50	M
*Atractus paucidens*	EPN 8730	–	–	–	–	–	–	–	–	–	246	53	M
*Atractus paucidens*	EPN 8731	–	–	–	–	–	–	–	–	–	237	51	M
*Atractus paucidens*	MHNG 2309.065	156	46	15	15	15	2	7	6	6	196	45	M
*Atractus paucidens*	MNHN 1906.245	186	40	17	17	17	2	7	7	–	262	42	M
*Atractus pyroni*	MZUTI 5107	143	16	15	15	15	1	6	5	8	443	34	F
*Atractus roulei*	QCAZ 6256	135	27	15	15	15	1	6	6	9	337	48	M
*Atractus roulei*	QCAZ 7887	146	25	15	15	15	1	5	6	9	309	39	M
*Atractus roulei*	QCAZ 7902	156	19	15	15	15	1	6	7	11	392	37	F
*Atractus roulei*	QCAZ 9643	149	17	15	15	15	1	6	6	11	139	13	F
*Atractus roulei*	QCAZ 9652	143	19	15	15	15	1	6	6	13	230	21	F
*Atractus savagei*	DHMECN 3800	166	25	17	17	17	2	6	7	7	214	23	F
*Atractus snethlageae*	MNHN 1906.244	151	29	17	17	17	2	7	7	7	283	35	F
*Atractus snethlageae*	MNHN 1994.1171	160	27	17	17	17	2	7	7	8	315	35	F
*Atractus touzeti*	ANF 2390	176	31	17	17	17	2	7	7	7	652	71	F
*Atractus trilineatus*	MNHN 1898.313	141	19	15	15	15	2	7	7	8	179	19	M
*Atractus trilineatus*	MNHN 1898.314	132	21	15	15	15	2	7	7	7	182	20	M
*Atractus typhon*	DHMECN 9632	153	47	15	15	15	2	7	6	7	187	31	M
*Atractus typhon*	FHGO 10438	166	41	15	15	15	2	7	7	6	370	68	M
*Atractus typhon*	FHGO 10439	158	48	16	16	16	2	7	7	7	349	87	F
